# Current Status: Site-Specific Antibody Drug Conjugates

**DOI:** 10.1007/s10875-016-0265-6

**Published:** 2016-03-22

**Authors:** Dominik Schumacher, Christian P. R. Hackenberger, Heinrich Leonhardt, Jonas Helma

**Affiliations:** Chemical Biology and Department of Chemistry, Leibniz-Institut für Molekulare Pharmakologie and Humboldt Universität zu Berlin, Berlin, Germany; Department of Biology II, Ludwig-Maximilians-Universität München and Center for Integrated Protein Science Munich, Planegg-Martinsried, Germany

**Keywords:** Antibody drug conjugates (ADCs), drug-antibody ratio (DAR), site-specific conjugation, therapeutic window, unnatural amino acids (UAA), monomethyl auristatin E/F (MMAE/MMAF), THIOMAB, microbial transglutaminase (MTG), formylglycine generating enzyme (FGE), Sortase A, tubulin tyrosine ligase (TTL), Tub-tag

## Abstract

Antibody drug conjugates (ADCs), a promising class of cancer biopharmaceuticals**,** combine the specificity of therapeutic antibodies with the pharmacological potency of chemical, cytotoxic drugs. Ever since the first ADCs on the market, a plethora of novel ADC technologies has emerged, covering as diverse aspects as antibody engineering, chemical linker optimization and novel conjugation strategies, together aiming at constantly widening the therapeutic window for ADCs. This review primarily focuses on novel chemical and biotechnological strategies for the site-directed attachment of drugs that are currently validated for 2nd generation ADCs to promote conjugate homogeneity and overall stability.

## Introduction

**Fig. 1:**
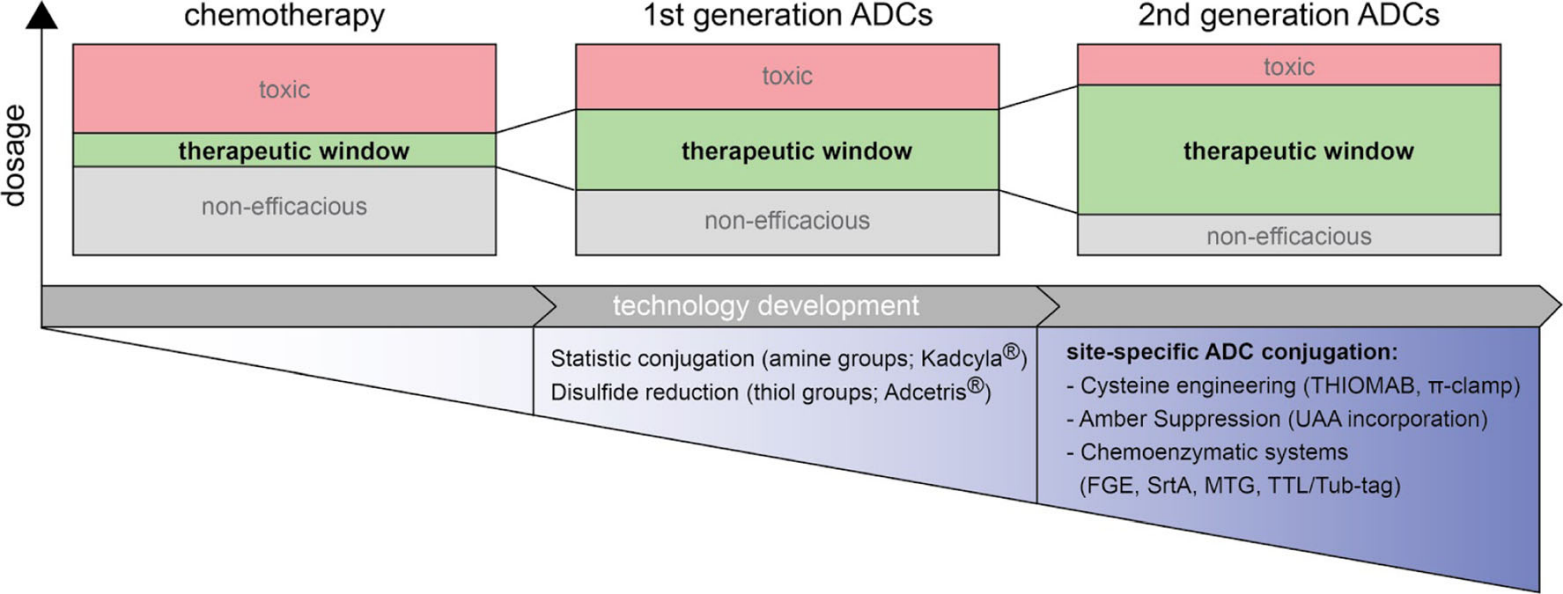
Novel ADC conjugation technologies for a widened therapeutic window. The antibody-mediated delivery of cytotoxic drugs at tumor sites lowers drug toxicity and enhances efficacy compared to conventional chemotherapeutics. Techniques for the conjugation of 1st generation ADCs are associated with conjugate heterogeneity issues. Thus, ADCs of the next generation are generated via site-directed conjugation approaches to improve the therapeutic activity

Chemotherapeutic strategies have long been used as the primary treatment against a broad range of cancers. However, tumor-cell specificity is only addressed with regard to rapid cell division rates present in most cancers, a feature that is true for a lot of non-malignant cell types as well, leading to systemic side-effects. Thus, targeted cancer treatments with therapeutic antibody biologics have gained major interest in the pharmaceutical and biopharmaceutical industry. In recent years however, huge efforts have been made to merge the positive features of chemical and biological cancer treatments with the development of antibody drug conjugates (ADCs) that deliver the highly cytotoxic drugs directly at the tumor site. As such, ADCs widen the therapeutic window in comparison to chemotherapeutics: The tumor-targeted, antibody-mediated drug delivery approach decreases the minimum effective dose and at the same time elevates the maximum tolerated dose (Fig. 1).

With such promising properties and the recent FDA approval of the first two ADCs, Kadcyla® and Adcetris®, the ADC field has gained momentum in all relevant directions with increasing knowledge about the major challenges, drawbacks and screws to turn for improving the quality and efficacy of modern cancer treatments covered by recent technology reviews [1–3] and currently over 40 ADCs in clinical trials [4].

Many choices have to be made for the successful generation and application of ADCs. The antibody defines the cellular target. To date various different molecular targets in both solid and haematological cancers are being exploited for ADC development [5]. While targeting surface antigens that are drastically overrepresented in malignant cells, as it is true for e.g. HER2 [6], is generally advantageous, ADCs targeting less selectively expressed cancer markers need to be carefully designed and fine-tuned to maintain the specific therapeutic effect. One such fine-tune parameter is the stoichiometric ratio of drug molecules per antibody molecule (Drug-antibody-ratio, DAR). ADC potency increases with increasing DAR, however plasma clearance accelerates as well [7], possibly due to increased ADC hydrophobicity derived from the conjugated payload [8, 9]. Thus, novel approaches to modulate ADC hydrophobicity and the corresponding ADC aggregation potential are highly desired [10]. Another important parameter affecting the therapeutic window of ADCs is related to the type of drug conjugation technique. Historically, cytotoxic payloads have been conjugated via natural antibody residues in a statistical fashion, leading to heterogeneous conjugate populations [11]. In contrast, the controlled, site-specific conjugation has the potential to overcome heterogeneity and widen the therapeutic window [12] (Fig. 1). In consequence, a variety of novel approaches for the site-specific drug conjugation has been developed over the last years. Site-specific technologies range from purely chemical methods, to genetic engineering and chemoenzymatic manufacturing. Here, we discuss current developments and promising future directions within this field.

### Statistic Conjugation

Mylotarg® (Gemtuzumab ozogamicin) targeting CD33 was the first ADC to be approved by the FDA for the treatment of myeloid leukemia in 2000 and statistically conjugated via surface exposed lysine residues of the antibody molecule. Ten years later, Pfizer voluntarily withdrew it from the US and European market due to low efficacy and possible toxicity observed in a second phase III study [13]. This may be attributed to the rather broad DAR of four to six and approximately 50 % unconjugated antibody being present in the product mixture [3, 14]. Kadcyla® is another FDA approved ADC where conjugation occurs at accessible lysine residues of the Trastuzumab IgG targeting HER2. The corresponding emtansine drug (DM1) is equipped with an amine-reactive succinimide ester group for conjugation. The Trastuzumab IgG contains in total 88 lysines and 70 out of these have been shown to be conjugated with DM1 [15]. While the DAR distribution may be narrowed to a rather constant level (between 3 and 4), 70 different conjugation sites result in a vastly complex mixture of ADC species with possibly divergent therapeutic and pharmacokinetic properties. Although Kadcyla® is successfully applied for the treatment of HER2-positive metastatic breast cancer, significant efforts are made to reduce the heterogeneity obtained by statistic conjugation methods and gain more control over the conjugation process.

### Site-Specific Chemical Conjugation to Cysteine Residues

**Fig. 2:**
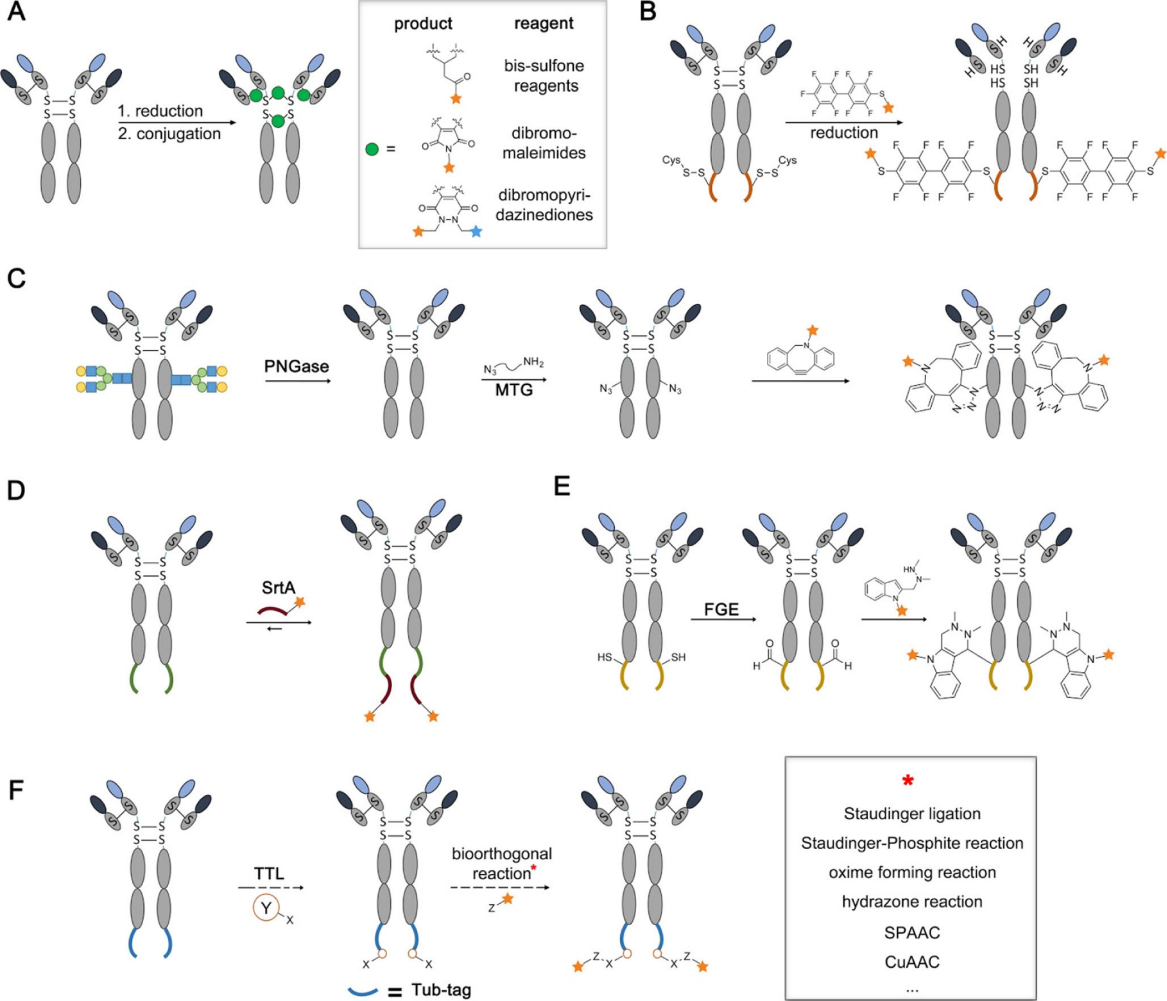
Techniques for the production of 2nd generation ADCs. **a** Re-bridging disulfides by bis-sulfone reagents, dibromomaleimides and dibromopyridazinediones reduces maximum DAR from eight to four. **b** The π-clamp (shown in *orange*) mediates site-specific conjugation with perfluoroaromatic reagents to its cysteine. **b** After removal of the antibody’s glycan by the enzyme PNGase, a transglutaminase is used to form an isopeptide bond between Q295 and an incoming amine-azide-linker. The drug molecule is attached to the azide by a strain promoted azide alkyne click reaction. **d** The transpeptidase Sortase A catalyzes the reversible formation of an amide-bond between threonine of the sequence LPXTG (shown in *green*) and a drug derivatives with a N-terminal penta-glycine motive (shown in *red*). **e** Formylglycine generating enzyme (FGE) transforms the thiol within the sequence CXPXR (shown in *yellow*) to an aldehyde. A following Pictet-Spengler type reaction generates stable conjugation-products. **f** Tub-tag labeling is a versatile and efficient tool to produce novel ADC derivatives. TTL mediates the site-specific attachment of various tyrosine derivatives to the C-terminus of the Tub-tag (shown in *blue*). In a second step, the cytotoxic drug is conjugated to the tyrosine derivatives using well established bioorthogonal chemistry (SPAAC = strain-promoted azide alkyne cycloaddition; CuAAC = copper-catalyzed azide alkyne cycloaddition)

The selective reduction of the four intermolecular disulfide bonds within an IgG molecule followed by maleimide-thiol conjugation to cytotoxic drugs was one of the first attempts to increase the control of the conjugation sites and to reduce the heterogeneity observed using lysine-based conjugation methods [16]. Adcetris®, the FDA approved CD30-specific antibody conjugated to monomethyl auristatin E (MMAE) for the treatment of hodgkin lymphoma, is the most prominent example of this strategy. The four disulfides bridging the IgG heavy and light chains yield eight potential conjugation sites inherently reducing conjugate heterogeneity, but still causing diverse ADC species with DAR distribution between zero and eight [17]. Moreover, maleimide conjugated ADCs are prone to hydrolysis and unwanted thiol exchange might result in attachment of the drug to endogenous proteins [18]. Using exocyclic olefinic maleimides might solve this problem, since their conjugated products are resistant towards thiol-exchange [19]. The companies Thiologics and Polytherics are using cysteine bridging dibromomaleimides and bis-sulfone reagents as conjugation scaffolds, respectively, to meet the challenges arising from instability and heterogeneity (Fig. 2a) [20, 21]. By re-bridging the reduced disulfides, they increase stability and reduce the maximum amount of cytotoxic drugs to be attached from eight to four. In a recent study, dibromopyridazinediones have been used to generate antibodies with two different payloads orthogonally attached giving rise to development of dual drug ADCs (Fig. 2a) [22].

A different approach to reduce heterogeneity of cysteine-based conjugated ADCs is the engineering of additional cysteine residues to the antibody leaving the interchain disulfides untouched [23, 24]. Initial difficulties arose from the oxidation of the mutated cysteines and the inability to selectively reduce them in the presence of the antibodies disulfides. These problems were not resolved until 2008, when Junutula and co-workers developed IgGs with two unpaired cysteines (called THIOMABs) and treated them with an optimized reduction/oxidation protocol in which all interchain disulfides are reinstalled after full reduction, leaving the unpaired cysteines unblocked for following cytotoxic drug attachment. By this, they were able to obtain homogeneous ADC conjugates (DAR of two) with comparable efficacy but significant reduction of in vivo toxicity, resulting in an improved therapeutic index [12]. Moreover, such flexible cysteine integration in combination with mathematical modelling allows the choice of a favorable intramolecular microenvironment and a defined DAR that positively affects the overall therapeutic activity of a given ADC [18, 25]. Neri and coworkers engineered a cysteine residue to the N-terminus of recombinant antibodies and used a thiazolidine linker to site-specifically conjugate the drug cemadotin. The thiazolidine hydrolyzes over time (half-life: 45 h in PBS 37 °C) releasing the free drug [26]. Very recently, Zhang et al. introduced a different approach to reduce the heterogeneity in cysteine-based ADC production. Perfluoroaromatic reagents are used to selectively conjugate payloads to the cysteine of a four amino acid sequence called π-clamp (FCPF). By this, they were able to conjugate a perfluoroaryl derivative of monomethyl auristatin F (MMAF) to Trastuzumab containing a C-terminal π-clamp whereas no reaction with conventional Trastuzumab was observed under the same reaction conditions (Fig. 2b) [27].

Cysteine mediated production of ADCs and the ideas arising thereof laid the foundation for site-specific conjugations of drugs to antibodies. Ever since it was shown that site-specific conjugation positively influences the characteristics of ADCs, the field has rapidly adapted and a significant number of novel techniques has emerged for drug loading at defined location within the antibody.

### Incorporation of Unnatural Amino Acids (UAAs)

Amber suppression pioneered by Schultz is the most frequently used method for the incorporation of unnatural amino acids to proteins and antibodies [28, 29]. The unnatural amino acid contains a bioorthogonal handle that enables the site-specific attachment of a payload in the presence of all naturally occurring functional groups. One of the major advantages of this technology is that the position of the unnatural amino acid, and therefore the conjugation site of the cytotoxic drug, can easily be varied by a single amino-acid mutation. Moreover, many different unnatural amino acids have successfully been incorporated to proteins by this method and one can choose from a broad selection of substrates increasing chemical flexibility [30]. In cooperation with the company Ambrx, Schultz and coworkers expressed Trastuzumab and a anti-5 T4 antibody in mammalian cells. By the use of an orthogonal tRNA/aminoacyl-tRNA pair and an amber stop codon (TAG) they placed *p*-acetylphenylalanine at distinct sites within the antibodies and aminooxy-auristatin F and auristatin D derivatives were conjugated by oxime ligation. Although the final conjugation step took up to 4 days at slightly acidic pH, the auristatin F conjugates had a similar pharmacokinetic profile in rats to that of unconjugated Trastuzumab. The auristatin D ADCs showed superior efficacy (in vitro and in rats) and in vivo pharmacokinetics compared to cysteine conjugated counterparts [31, 32]. One of the major difficulties of using amber suppression for the production of ADCs is the high technical demand and relatively low antibody titers in the range of 300–1000 mg/L [31, 32]. Moreover, the amber stop codon usage in mammalian cells is relatively high resulting in a heterogenous mixture of antibodies with falsely incorporated natural amino acids, that may lead to toxic side-effects and makes purification of the ADCs much more complicated. Sutro Biopharma tries to sort out this problem by using an *E. coli* based cell-free system for the production of heterogenous ADCs. However, the antibody titer that they were able to achieve by this was even lower (250 mg/L) and all antibodies generated by cell-free expression systems are not glycosylated [33]. Even though several unglycosylated antibodies are evaluated for human therapy [34], the impact of removing this posttranslational modification is still under debate.

### Enzymatic and Chemoenzymatic Approaches

Within the last years, several enzymatic and chemoenzymatic methods for the site-specific functionalization of antibodies have been developed, among them the use of engineered glycotransferases, transglutaminases, the bacterial derived formyl glycine generating enzyme (FGE) and the transpeptidase Sortase A. In nature glycotransferases promote the transfer of an activated glycosyl donor to a lipid or glycoprotein and thereby play important roles in the posttranslational modification of proteins and in signaling pathways. Qasba et al. were able to show, that by the mutation of a few amino acids within the binding pocket of the beta-1,4-galactosyltransferase I, unnatural monosaccharides that carry a unique reactive group (a bioorthogonal handle) are accepted as substrates and can thereby be incorporated to glycan modified proteins [35]. Ever since, this knowledge has been used to site-specifically conjugate fluorophores, biotin-derivatives and cytotoxic drugs to antibodies [36–38]. In 2014, Sanofi-Genzyme published a combinatorial approach of beta-1,4-galactosyltransferase I and alpha-2,6-sialyltransferase to place a terminal sialic acid residue to the native glycosylation site of Trastuzumab and anti FAB. Subsequent mild periodate oxidation of the sialic acids and oxime ligation enabled site-specific conjugation of 1.6 molecules of monomethyl auristatin E (MMAE) or Dol10 to the antibodies, resulting in a comparable antitumor activity with a significantly lower toxin loading to that of statistically conjugated ADCs [39]. Enzymatic glycosylation approaches are promising and straightforward strategies to equip antibodies with cytotoxic drugs without the need of antibody engineering. However, since glycosylation is a heterogeneous modification, producing homogenous ADCs by this technology is very complicated. Moreover, changing the glycosylation pattern of the antibodies may lead to an immunogenic response in humans which was already shown for several unnatural sialic acid derivatives [40, 41].

In 2010, the Schibli group was showed site-specific functionalization of IgGs (rituximab and the anti-L1-CAM chCE7) using an amide bond forming transglutaminase. After removal of the antibodies N-glycan using PNGase, various amine-containing substrates were coupled to Q295 by isopeptide bond formation [42]. Using a chemoenzymatic two-step variant of the transglutaminase technology, they were able to produce homogenous Trastuzumab-MMAE conjugates with a DAR of 2 functionalized at Q295 (Fig 2c) [43]. Introducing transglutaminase recognition sequences into the antibody is another approach that does not rely on PNGase pretreatment. Rinat-Pfizer placed the amino-acid tag LLQA to several positions to the heavy and light chain of anti-EGFR, anti-HER2 and anti-M1S1 antibodies and conjugated fluorophores and auristatin derivatives using a transglutaminase from *Streptoverticillium mobaraense* resulting in DAR of 1.2–2 [44]. These conjugates showed a larger therapeutic window in rats compared to heterogenous cysteine-conjugated derivatives with a DAR of 3.6. However, careful MS analysis revealed unspecific reactivity of the transglutaminase at Q295 making a Q295N mutation necessary to ensure specific reactivity [45]. The Group of Kolmar designed a novel transglutaminase recognition tag based on the crystal structure of a natural protein substrate of a bacterial transglutaminase. Placing this structural constraint tag to the C-terminus of the heavy chain of cetuximab allowed fast and efficient conjugation to amine containing biotins [46]. However, to prevent intramolecular crosslinking of the antibody catalyzed by the enzyme, the terminal K447 had to be removed and the amount of Q295 site-reactivity remains unknown.

The transpeptidase Sortase A (*Staphylococcus aureus*) has intensively been studied for the site-specific modification of proteins and antibodies [47–49]. The thiol of the enzyme’s C148 attacks the amide bond between threonine and glycine of the recognition sequence LPXTG placed at the terminus of a protein of choice. This leads to the release of the terminal glycine and formation of a thioacyl intermediate. A following nucleophilic attack of another glycine-peptide equipped with a cargo of choice results in the reformation of an amide-bond and thereby the site-specific functionalization of the protein. The reversibility of transpeptidase mediated reactions is a major drawback of this technology since it reduces efficiency and increases the need of tedious post-reaction purification. This has been addressed to a certain extent by the use of depsipeptide substrates [50] and enhanced Sortase A variants [51]. The company NBE-Therapeutics AG developed a Sortase A based platform called SMAC-technology (sortase mediated antibody conjugation technology) to generate various ADCs with different linkers and toxins (MMAE and DM1) [52]. By the C-terminal addition of a 14 or 19 amino acid long peptide sequence containing a LPETG and a C-terminal Strep II tag to the heavy and light chain of the antibodies trastuzumab and cAc10 they were able to achieve DAR ratios of 3.05–3.53 (Fig. 2d). Moreover, since the Strep II tag is only present in unreacted starting material, they tremendously simplified post-reaction purification. Even though the SMAC derived ADCs contained higher aggregate content compared to the unmodified counterparts, they were sufficient for in vitro and in vivo studies and showed similar potency compared to their chemically modified counterparts adcetris and kadcyla.

Another chemoenzymatic strategy, in which a formylglycine generating enzymes (FGE) oxidizes the cysteine side chain of the peptide sequence CXPXR to a formylglycine was pioneered and developed in the labs of Diercks and Bertozzi [53, 54]. Oxime forming reactions or, to gain hydrolytically stable products, Pictet-Spengler type reactions [55–56] are used to conjugate payload to this bioorthogonal group. The company Redwood Bioscience (now Catalent Pharma solutions) uses this approach to obtain side-specific ADCs. They introduced the CXPXR sequence into eight sites of trastuzumab, out of which three were identified to be suitable for maytansinoid conjugation (Fig. 2e). The plasma stability, in vivo half-life and efficacy in a xenograft mice model were dependent on the conjugation site and showed the best values for conjugates that were functionalized at the C-terminus of the heavy chain. Moreover these conjugates showed an improved safety profile to conventionally conjugated lysine ADCs [57]. Since prokaryotic and eukaryotic FGEs tend to precipitate, they recently published a optimized protocol to obtain soluble FGEs and showed that FGEs are metalloenzymes and their activity dependent on Cu(II) activation [58].

### Tub-tag Labeling: An Emerging Approach for ADC Conjugation

Tub-tag labeling is a novel approach for the site-specific modification of antibodies that combines the above mentioned use of UAA incorporation with a highly efficient chemoenzymatic system [59]. The technique is based on the enzyme tubulin tyrosine ligase (TTL) that is naturally involved in the intracellular regulation of microtubule stability [60]. TTL recognizes a 14 amino acid recognition motif at the C-terminus of alpha-tubulin and posttranslationally attaches a terminal tyrosine residue [61]. When recombinantly fused to an antibody, the recognition motif (Tub-tag) allows the TTL-mediated attachment of unnatural tyrosine derivatives that carry uniquely reactive groups for chemoselective conjugation such as strain-promoted alkyne azide cycloadditions (SPAAC, Fig. 2f). The method has shown labeling efficiencies up to 99 % in vitro and is compatible with a broad range of established conjugation chemistries (Fig. 2f). While the functional effect of the human-derived peptide at the C-terminus of therapeutic antibodies remains to be validated, its glutamate-rich, strongly hydrophilic character may well provide a potential intrinsic measure to counteract described drug and linker associated hydrophobicity and aggregation issues [8, 9]. Thus, it provides a potentially favourable microenvironment for drug loading and enhanced DAR flexibility, thereby meeting two of the most relevant needs for modern ADC conjugation.

## Conclusions

The still increasing number of ADCs in clinical trials and the constantly growing number of ADC-related publications do underline the significance of the ‘magic bullet’ oncology approach that has first been postulated in 1908 by Paul Ehrlich. Nonetheless, the field has witnessed a number of recent pitfalls that exposed the major technological vulnerabilities where improvement is needed. Most of these weak spots revolve around ADC efficacy, toxicity, clearance and stability and are currently tackled from various directions. This includes the identification of novel drugs, antibody engineering to identify regions that are well suited for drug attachment and versatile chemical linker strategies to modulate the drug load, release and overall ADC stability.

Moreover, novel site-specific coupling strategies to allow for homogeneous ADC species with defined and controllable therapeutic properties are under investigation. All existing site-specific conjugation strategies have advantages and disadvantages and negotiate scientific parameters such as the extent of antibody manipulation and in consequence its functional integrity, conjugation efficiencies, conjugate stability, chemical versatility as well as applied ‘real world’ parameters such as technical handling for cost-effective, industrial ADC production.

With an ever increasing portfolio of ADC enabling technologies at hand that together allow for better, versatile and tailor made conjugates, probably one of the most promising and pressing tasks within the ADC field lies in the search for novel antibody conjugate indications beyond classical oncology targets. This particularly includes pathogens that have previously been hard or impossible to tackle, as has recently been exemplified for novel Antibody-antibiotic conjugates targeting well-hidden, intracellular populations of pathogenic bacteria [62].
